# Author Correction: Omicron BA.4/BA.5 escape neutralizing immunity elicited by BA.1 infection

**DOI:** 10.1038/s41467-022-33371-0

**Published:** 2022-10-13

**Authors:** Khadija Khan, Farina Karim, Yashica Ganga, Mallory Bernstein, Zesuliwe Jule, Kajal Reedoy, Sandile Cele, Gila Lustig, Daniel Amoako, Nicole Wolter, Natasha Samsunder, Aida Sivro, James Emmanuel San, Jennifer Giandhari, Houriiyah Tegally, Sureshnee Pillay, Yeshnee Naidoo, Matilda Mazibuko, Yoliswa Miya, Nokuthula Ngcobo, Nithendra Manickchund, Nombulelo Magula, Quarraisha Abdool Karim, Anne von Gottberg, Salim S. Abdool Karim, Willem Hanekom, Bernadett I. Gosnell, COMMIT-KZN Team, Thandeka Khoza, Theresa Smit, Emily Wong, Richard J. Lessells, Tulio de Oliveira, Mahomed-Yunus S. Moosa, Alex Sigal

**Affiliations:** 1 Africa Health Research Institute, Durban, South Africa; 2 School of Laboratory Medicine and Medical Sciences, University of KwaZulu-Natal, Durban, South Africa; 3 Centre for the AIDS Programme of Research in South Africa, Durban, South Africa; 4 National Institute for Communicable Diseases of the National Health Laboratory Service, Johannesburg, South Africa; 5 School of Health Sciences, College of Health Sciences, University of KwaZulu-Natal, Durban, South Africa; 6 School of Pathology, Faculty of Health Sciences, University of the Witwatersrand, Johannesburg, South Africa; 7 Department of Medical Microbiology, University of KwaZulu-Natal, Durban, South Africa; 8 KwaZulu-Natal Research Innovation and Sequencing Platform, Durban, South Africa; 9 Centre for Epidemic Response and Innovation (CERI), School of Data Science and Computational Thinking, Stellenbosch University, Stellenbosch, South Africa; 10 Department of Infectious Diseases, Nelson R. Mandela School of Clinical Medicine, University of KwaZulu-Natal, Durban, South Africa; 11 Division of Internal Medicine, Nelson R. Mandela School of Medicine, University of Kwa-Zulu Natal, Durban, South Africa; 12 Department of Epidemiology, Mailman School of Public Health, Columbia University, New York, NY USA; 13 Division of Infection and Immunity, University College London, London, UK; 14 Department of Global Health, University of Washington, Seattle, WA USA; 15 Max Planck Institute for Infection Biology, Berlin, Germany; 16 Division of Infectious Diseases, University of Alabama at Birmingham, Birmingham, AL USA

**Keywords:** Antibodies, Viral infection, SARS-CoV-2

Correction to: *Nature Communications* 10.1038/s41467-022-32396-9, published online 10 August 2022

The original version of the Supplementary Information associated with this Article contained errors. Supplementary Table S1 was omitted. The title of Supplementary Fig. S1 incorrectly read ‘BA.4 and BA.4’, rather than the correct ‘BA.4 and BA.5’. The HTML has been updated to include a corrected version of the Supplementary Information.

